# A Purification Strategy Utilizing Hydrophobic Interaction Chromatography to Obtain Homogeneous Species from a Site-Specific Antibody Drug Conjugate Produced by AJICAP™ First Generation

**DOI:** 10.3390/antib9020016

**Published:** 2020-05-18

**Authors:** Yutaka Matsuda, Monica Leung, Tatsuya Okuzumi, Brian Mendelsohn

**Affiliations:** 1 Ajinomoto Bio-Pharma Services, 11040 Roselle Street, San Diego, CA 92121, USA; Yutaka.Matsuda@US.AjiBio-Pharma.com (Y.M.); Monica.Leung@US.AjiBio-Pharma.com (M.L.); 2 Ajinomoto Co., Inc., 1-1 Suzuki-cho, Kawasaki-ku, Kawasaki, Kanagawa 2108681, Japan; tatsuya_okuzumi@ajinomoto.com

**Keywords:** antibody drug conjugate, site-specific conjugation, hydrophobic interaction chromatography (HIC) purification, AJICAP™

## Abstract

In recent years, site-specific antibody drug conjugates (ADC)s have been in great demand because they have an expanded therapeutic index compared with conventional ADCs. AJICAP™ technology is a chemical conjugation platform to obtain site-specific ADCs through the use of a class of Fc-affinity compounds. Promising results from early technology development studies led to further investigation of AJICAP™ ADC materials to obtain site-specific and homogeneous drug antibody ratio (DAR) ADCs. Here we report site-specific conjugation followed by a preparative hydrophobic interaction chromatography (HIC) purification strategy to obtain purified “DAR = 1.0” and “DAR = 2.0” AJICAP™ ADC materials. Optimization of the mobile phase conditions and resin achieved a high recovery rate. In vitro biological assay demonstrated the target selective activity for purified homogeneous DAR ADCs. These results indicate the ability of a HIC purification strategy to provide “DAR = 1.0” and “DAR = 2.0” AJICAP™ ADCs with considerable potency and target selectivity.

## 1. Introduction

Recent years have seen rapid growth in the research and development of antibody drug conjugates (ADCs), with 7 ADCs approved for clinical use by the Food and Drug Administration (FDA) and over 85 ADCs currently in clinical development [1,2,3]. All of the current ADCs on the market have a stochastic distribution of cytotoxic drugs conjugated to multiple sites of the antibody, leading to variation between batches in both the drug to antibody ratio (DAR) and the drug-linker attachment sites [4]. These heterogeneous ADCs produced by nonspecific conjugation techniques may have diminished efficacy or increased toxicity, limiting their therapeutic index compared with a homogeneous ADC [5,6].

Several advancements have been made regarding site-specific conjugation technologies in efforts to overcome the issues derived from the heterogeneity of traditional ADCs [7]. Our research group has developed an innovative chemical site-specific conjugation platform termed AJICAP™ first generation [8]. This technology enables the installation of reactive moieties compatible with payloads and linkers, such as thiol functional groups to well-defined amino acid residues through the use of Fc-affinity peptide reagents. Affinity peptides react with a target lysine in the Fc region of antibodies to produce antibody-peptide conjugates. Release of the peptide from the antibody by linker cleavage results in site-specific thiol-modified antibodies. These thiol-modified antibodies easily react with a variety of maleimide-containing drug-linkers to provide site-specific DAR = 2 ADCs. Early evaluation studies, including in vitro and in vivo assays and a surface plasmon resonance study, indicated that an AJICAP™-ADC retained target selectivity and displayed expected ADC potency. AJICAP™ technology platform has been also reported to enable a reliable and robust gram-scale synthesis of site-specific ADCs while adhering to good manufacturing practices [9,10]. Peptide mapping analysis of an AJICAP™-ADC has proved the site specificity by revealing that the conjugation position was solely at Lys 248 [11]. However, all site-specific conjugation methodologies, including our AJICAP™ technology, have not been able provide truly homogeneous ADCs in terms of the DAR, but rather only low-heterogeneity ADCs that contain small amounts of unreacted and unpurified species.

In contrast to conjugation methodologies, literature reports examining purification techniques to separate DAR species have been extremely limited to date [12]. In 2004, a group at Seattle Genetics compared the efficacy and pharmacokinetics of stochastic ADCs with DAR = 2, DAR = 4, and DAR = 8 purified by preparative hydrophobic interaction chromatography (HIC) [13]. This report indicated that decreasing the drug loading per antibody enhanced the therapeutic index, which motivated an investigation to obtain ADCs with DAR = 2.

**Figure 1:**
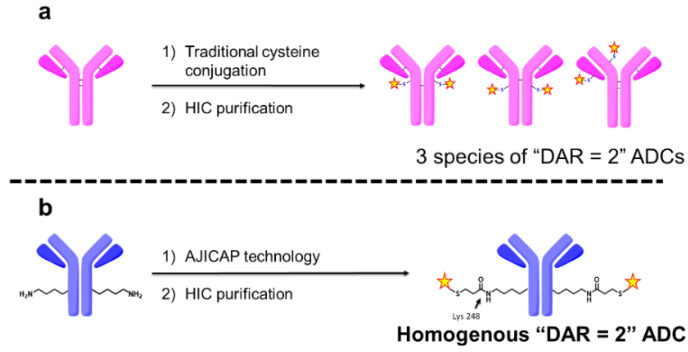
Comparison of two different antibody drug conjugates (ADC) synthetic methods that include conjugation and preparative hydrophobic interaction chromatography (HIC) purification: (**a**) Traditional cysteine conjugation approach and (**b**) AJICAP™ approach.

This report described the purification of an ADC with a particular DAR, but not a homogeneous site-specific ADC as the group used traditional cysteine-based conjugation, which led to a distribution of drug-linker attachment sites. For example, theoretically there are three species of ADC with DAR = 2 which could be produced by this approach, as shown in Figure 1a. In 2014, Goodwin and coworkers bypassed the disadvantage of stochastic cysteine-based ADCs using a cysteine rebridging approach [14]. This group also conducted HIC purification to separate several DAR species of cysteine rebridged ADCs, enabling control of the DAR value [15]. However, the technique used in this work was limited to create ADCs with DAR = 4. Furthermore, the methodology used was discovered to produce some undesired misbridged ADCs [16]. Obtaining an ADC with DAR = 2 is the initial target for the application of our groups’ site-specific conjugation technology to increase the ADC therapeutic index [17].

**Figure 2:**
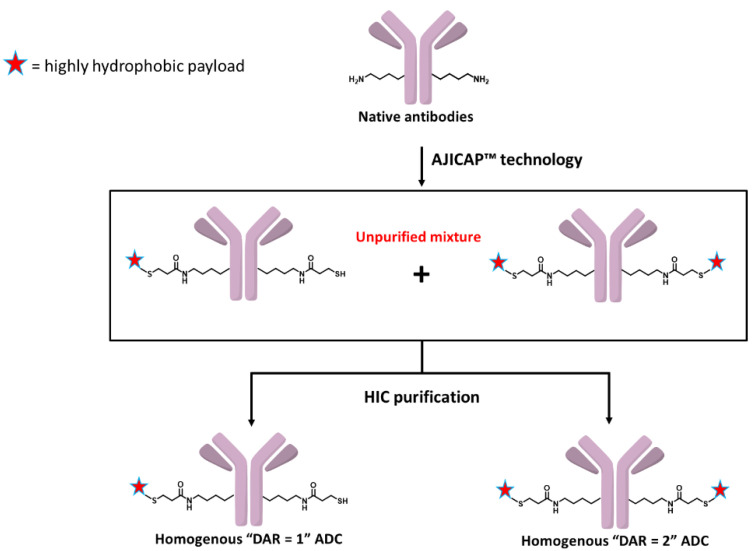
Overview of site-specific conjugation/HIC purification strategy.

The stochastic distribution of traditional ADCs is known to have a negative effect on the therapeutic index. In 2013, Rajpal, Strop, and coworkers reported that the conjugation site has a considerable impact on the stability and properties of ADCs [17]. These results indicated that an ADC with a single conjugation site is preferable to expand the therapeutic index, leading us to believe that a site-specific and homogeneous ADC with DAR = 2 could be an ideal ADC format. Therefore, we attempted to obtain homogeneous ADCs with a particular DAR and a single conjugation site produced by a combination of our chemical site-specific conjugation technology termed “AJICAP™” and preparative HIC purification, as shown in Figure 2.

In addition to creating ADC with DAR = 2, we are interested in developing methodologies to produce homogeneous ADCs with DAR = 1, which have the potential to expand the therapeutic index of hydrophobic or highly potent payload-based ADCs. In recent years, highly potent payloads such as pyrrolobenzodiazepine (PBD) dimers have received a large amount of attention [18]. The mode of action and high potency of PBD dimers makes these payloads of interest in the synthesis of ADCs; however, the high potency is also a double-edged sword because of the risk of premature payload-release or other off-target toxicities resulting in possible dose limiting toxicities. Several PBD-based ADCs have been discontinued during clinical trials [19] and we speculated that the reasons behind these discontinuations is likely due to a narrow therapeutic window. A homogeneous ADC with a low DAR (DAR = 1) is a possible solution that may result in a clinically relevant therapeutic window for PBD-based protein conjugates. The combination strategy we describe herein can produce for evaluation both ADCs with DAR = 2 and ADCs with DAR = 1 conjugated in a site-specific manner, and the biological studies using homogenous site-specific ADCs can be referred to for guidance to support our hypothesis. Here we report our current efforts investigating a combination strategy to obtain purified homogeneous “DAR = 1” and “DAR = 2” site-specific ADCs. Biological evaluation of these purified homogeneous ADCs using cell-based assays was conducted to compare their efficacy and target selectivity.

This approach demonstrated a robust methodology combining site-specific conjugation and preparative HIC purification to obtain “ideal” homogeneous site-specific ADCs.

## 2. Materials and Methods

### 2.1. Materials

Human IgG1, trastuzumab (Herceptin^®^) was purchased from Roche Pharmaceutical Company (Switzerland). Maleimide-C6-valine-citrulline-monomethyl auristatin E (CAS#: 646502-53-6; MC-VC-MMAE) was purchased from Abzena (Bristol, PA, USA). The peptide reagent (1) was provided by Ajinomoto Co., Inc. All other chemicals were purchased from Sigma-Aldrich (St. Louis, MO, USA). All cell lines (HCC-1954 and NCI-N87 cancer cell lines) were purchased from ATCC (Gaithersburg, MD, USA).

### 2.2. Synthetic Procedure for Unpurified Trastuzumab-AJICAP™-MMAE

Unpurified trastuzumab-AJICAP™-MMAE (4) was synthesized according to our previous procedure [20]. Peptide reagent (1) was reacted with trastuzumab to produce the trastuzumab-peptide conjugate (*2*). Treatment of *2* by Tris (2-carboxyethyl)phosphine (TCEP) clipped off peptide moiety from *2* involving cleavage of interchain disulfide bonds. The resulting linker cleavage product was converted to product (*3*) by dehydroascorbic acid (DHAA) oxidation. Conjugation of 3 to MC-VC-MMAE was performed to produce trastuzumab-AJICAP™-MMAE (4A). Next, the buffer exchange of this solution was conducted by an Amicon Ultra centrifuge filter (10 kDa MWCO) to afford trastuzumab-AJICAP™-MMAE (4A) in formulation buffer (20 mM histidine containing 5% trehalose, pH 5.2).

### 2.3. Preparative HIC

Unpurified trastuzumab-AJICAP™-ADC (4A) was purified by HIC column (0.8 × 10 cm, 5 mL), prepacked by ToyoPearl Phenyl-650S HIC resin (Tosoh Bioscience) and attached to an AKTA Pure system [16]. All process was performed at room temperature. Before sample injection, the column was equilibrated with 3 column volumes of buffer A (50 mM sodium phosphate pH 7.0, 2 M sodium chloride (NaCl). For loading onto the column, 2.5 mL of unpurified ADC (20 mg/mL in formulation buffer) was mixed with 2.5 mL of buffer A. This resulting mixture (total 5 mL) was injected into the column and eluted using a linear gradient from 100% buffer to 100% buffer B (50 mM sodium phosphate pH 7.0, 20% isopropyl alcohol (IPA) v/v).

### 2.4. ADC Concentration

The concentration of ADCs was determined by the slope spectroscopy method with a SoloVPE system as previously reported [8]. Recovery of ADCs from purification was also calculated from ADC concentration.

### 2.5. HIC-HPLC Analysis

HIC-HPLC analysis was performed based on previously reported [21]. Each ADC was analyzed using Tosoh Bio Butyl-NPR 2.5 µm 4.6 × 35 mm column (Tosoh Bioscientific), connected to an Agilent 1260 HPLC system containing a binary gradient pump, a temperature-controlled column compartment, an autosampler, and a diode array detector. The system conditions were as follows: flow rate = 0.8 mL/min at 30 °C; mobile phase A (MPA) = 1.2 M (NH_4_)_2_SO_4_, 25 mM NaHPO_4_/NaH_2_PO_4_ (pH 6.0); mobile phase B (MPB) = 25 mM NaHPO_4_/NaH_2_PO_4_ (pH 6.0) with 25% IPA v/v). The absorbance was monitored at 280 nm (reference wavelength at 450 nm). Each ADC (1 mg/mL, 40 μL) was injected into the system and eluted over a 27 min run consisting of a 2 min isocratic hold at 0% MPB, a 20 min linear gradient from 0% to 100% MPB, a 2 min wash using 100% MPB, and a 3 min re-equilibration at 0% MPB.

### 2.6. RP-HPLC Analysis

RP-HPLC analysis was performed based on previously reported [21]. Each sample was prepared as follows: 1.0 mg/mL of ADCs in 500 mM tris buffer, pH 8.0, was diluted to 0.6 mg/mL in 8 M guanidine HCl and reduced by the addition of 1 M DL-Dithiothreitol (DTT). The mixture was incubated at 80 °C for 10 min and was analyzed using AdvanceBio RP-mAb Diphenyl, 2.1 × 100 mm, 3.5 μm column (Agilent), connected to an Agilent 1260 HPLC system containing a binary gradient pump, a temperature-controlled column compartment, an autosampler, and a diode array detector. The system conditions were as followed: flow rate = 0.4 mL/min at 70 °C; MPA = 0.1% trifluoroacetic acid (TFA) and 2% acetonitrile in water; mobile phase B (MPB) = 0.1% TFA in acetonitrile. The absorbance was monitored at 280 nm (reference wavelength at 450 nm). Each ADC (0.6 mg/mL, 20 μL) was injected into the system and eluted over a 35 min run consisting of a 2 min isocratic hold at 30% MPB, a 22 min linear gradient from 30% to 48% MPB, a 3 min wash using 95% MPB, and a 8 min re-equilibration at3 0% MPB. 

### 2.7. Q-TOF MS Analysis

Quadrupole time-of-flight mass spectrometry (QTOF MS) analysis was performed on a PLRP-S 2.1 × 50 mm, 1000 Å, 5 μm column (Agilent), connected to an Agilent 1260 HPLC system with an Agilent 6550 QTOF system containing a binary gradient pump, a temperature-controlled column compartment, and an autosampler. The elution conditions were as follows: mobile phase A = 0.1% formic acid, 0.01%, trifluoroacetic acid (TFA), 2% acetonitrile in water; mobile phase B = 0.1% formic acid and 0.01% TFA in acetonitrile; gradient 0–2 min isocratic hold at 20% MPB; 2–12 min, 20–50% B; 12–15 min, 95% B, 15–15 min, 20% B; flow rate = 0.3 mL/min. The absorbance was measured at 280 nm. Automatic data processing was performed with MassHunter BioConfirm software (Agilent) to analyze the intact and reduced MS spectra. For intact deconvolution, we used a mass range of 100,000–180,000 and a limited *m*/*z* range of 1000–4000. For reduction deconvolution, we used a mass range of 20,000–60,000 and a limited *m*/*z* range of 1000–3000. Moreover, we used DAR Calculator software (Agilent) to determine the PAR and DAR.

### 2.8. SEC-HPLC Analysis

Size exclusion chromatography (SEC)-HPLC analysis was performed based on previously reported [22]. Each ADCs was analyzed AdvanceBio SEC 300 Å, 4.6 × 150 mm, 2.7 µm column (Agilent), (Tosoh Bioscientific), connected to an Agilent 1260 HPLC system containing a binary gradient pump, a temperature-controlled column compartment, an autosampler, and a diode array detector. The system conditions were as follows: flow rate = 0.25 mL/min at 30 °C; mobile phase A (MPA) = 100 mM NaHPO_4_/NaH_2_PO_4_, 250 mM NaCl, 10% v/v IPA, pH 6.8 (mobile phase, MP). The absorbance was monitored at 280 nm (reference wavelength at 450 nm). Each ADC (1 mg/mL, 40 μL) was injected into the system and eluted over an 11 min run consisting of MP.

### 2.9. In Vitro Cytotoxicity

Trastuzumab-AJICAP™-ADCs (unpurified, DAR = 0, DAR = 1, and DAR = 2) and trastuzumab were analyzed using PC3, HCC-1954, and NCI-N87 cancer cell lines as previously reported [10].

## 3. Results and Discussion

### 3.1. Site-Specific Conjugation and Purification of Trastuzumab-AJICAP™-MMAE

**Figure 3:**
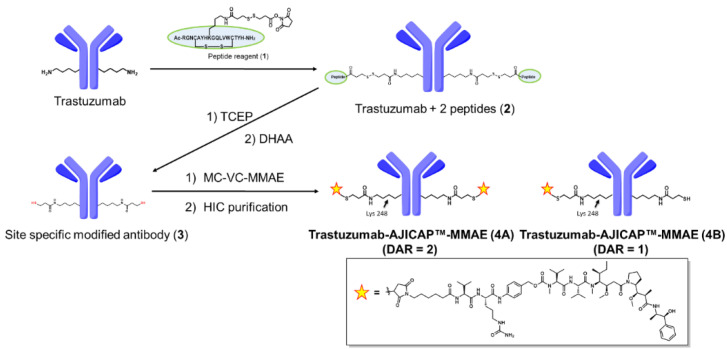
Site-Specific trastuzumab-AJICAP™-MMAE synthesis.

Site-specific trastuzumab-AJICAP™-MMAE was synthesized by the first generation AJICAP™ procedure (a well-established approach that in this case generated a defect-DAR ADC mixture), which provided gram-scale ADC materials shown in Figure 3 used in the present purification study. [20] The affinity peptide reagent (1) was reacted with Lys 248 in the Fc region of trastuzumab to provide trastuzumab-peptide conjugates (2) followed by a linker cleavage reaction to produce the thiol-incorporating antibody (3). Commercially available MC-VC-MMAE was linked to these newly formed thiol groups to afford an AJICAP™-ADC (4A + 4B). This site-occupancy was determined by peptide mapping analysis with denaturing RP-HPLC chromatography post trypsin digestion [11].

**Figure 4:**
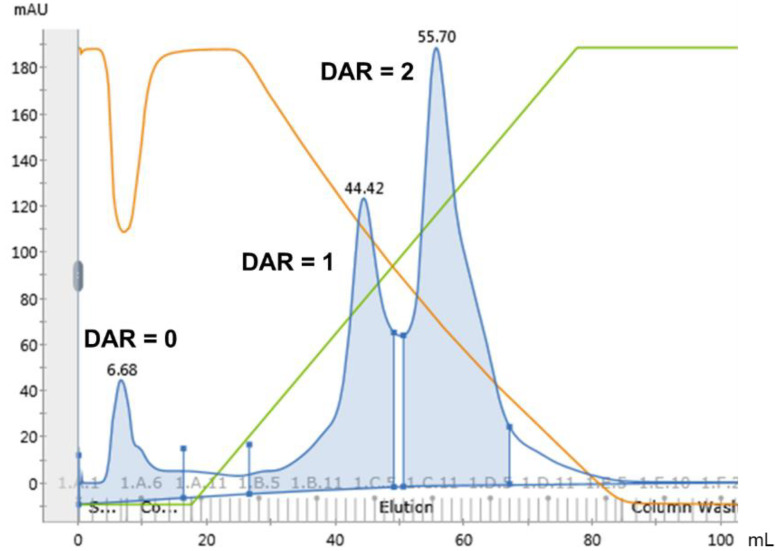
Chromatogram results of a Toyopearl Phenyl-650S column used for separation of different drug antibody ratio (DAR) species of unpurified trastuzumab-AJICAP™-MMAE (4). Blue line and area: UV_280 nm; green line: concentration of mobile phase B; orange line: conductivity.

Next, preparative HIC purification of the resulting AJICAP™-ADC was conducted with an AKTA Pure chromatography system. For the purification investigation, prepacked columns of various resins (purchased from Tosoh bioscience and listed in Table 1) were used. We first attempted using ammonium sulfate, which is one of the most common Hofmeister salts used to capture ADC compounds [12]. However, this strong lyotropic salt caused lower recovery of the ADC species. In comparison to ammonium sulfate, sodium chloride (NaCl) is a relatively weaker lyotropic salt, therefore 2 M NaCl in 50 mM sodium phosphate buffer (pH 7.0) was used as the capture buffer (mobile phase A) [13,14]. For the elution buffer (mobile phase B), IPA was expected to provide good separation of species with different DARs based on our previous analytical comparison study [20], hence we selected 20 v/v% IPA in 50 mM sodium phosphate buffer (pH 7.0). Resin screening was also conducted with this buffer composition, as shown in Table 1. In the case of trastuzumab-AJICAP™-MMAE, a Toyopearl Phenyl-650S column provided separated the DAR species, as shown in Figure 4. The DAR = 0 compound, which has low hydrophobicity, could not be captured by these purification conditions and passed through just after sample loading. However, DAR = 1 and DAR = 2 ADCs were captured by the HIC resin and eluted as the gradient proceeded. Lower DAR compounds, especially DAR = 0 can affect the efficacy of ADCs because these compounds may compete by binding to limited expressed target-antigen. Therefore, the removal of DAR = 0 material is important for producing efficacious ADCs. Our purification conditions, which can easily remove DAR = 0 species from a heterogeneous ADC mixture, have the potential to enhance the efficacy of other nonhomogeneous ADCs contaminated by DAR = 0 species. We also performed resin screening analysis with three different types of resins to determine the optimal resin. A butyl-type resin, which is more hydrophobic than a phenyl resin, did not elute the AJICAP™-ADC species even with the use of 30 v/v% IPA in the mobile phase B, as shown in Table 1. Conversely, a polypropylene glycol (PPG) resin, which contains a more hydrophilic column packing, provided no DAR separation. Therefore, we determined the optimal resin to be the phenyl resin, which allowed for the separation of compounds with different DARs and a moderate recovery. These results demonstrated the importance of the choice of an appropriate combination of column resin and buffer composition depending on the physical and chemical properties of the target ADCs.

The reproducibility of the present purification conditions using phenyl 650S resin (Entry 1 in Table 1) was confirmed at different sample scales (12, 20, and 50 mg). The recovery rate and the separation efficiency of the DAR were comparable at all three different scales.

**Figure 5:**
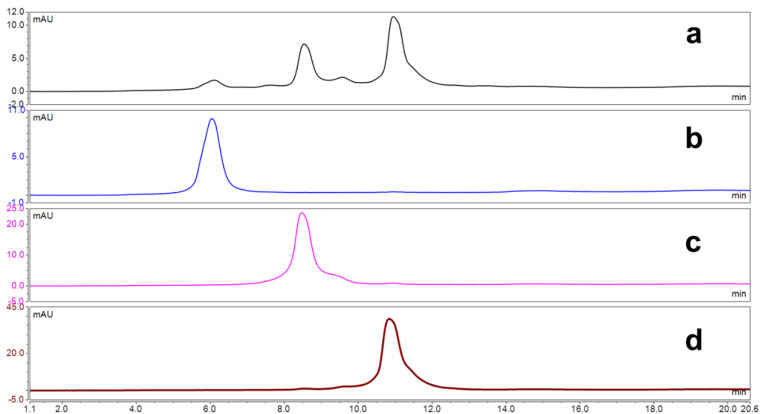
HIC analysis for DAR determination: (**a**) Unpurified ADC 4 (DAR = 1.6); (**b**) site-specifically modified antibody (3) after preparative HIC purification; (**c**) DAR = 1 trastuzumab-AJICAP-MMAE after preparative HIC purification; and (**d**) DAR = 2 trastuzumab-AJICAP-MMAE after preparative HIC purification.

DAR analysis was conducted by HIC as shown in Figure 5. After preparative HIC purification, homogeneous DAR species were observed. Q-TOF MS analysis supported these HIC results showing the homogeneity of several ADC compounds (Supplementary Materials Figures S1–S8). In a previous study, Q-TOF results completely matched with HIC analysis in the case of purified ADC due to its simple ADC composition [20]. Our present study further supports that result. RP-HPLC analysis was also carried out for DAR determination (Supplementary Materials Figures S9–S12), however slightly lower DAR results were obtained. It has been reported in a previous paper that the DAR value from RP-HPLC may be slightly lower than that measured from HIC analysis [10,20,23]. RP-HPLC analysis requires relatively high temperature and denaturing pretreatment with a combination of urea and DTT. These measurement conditions may have potential risks for degradation of ADC species which may result in lower DAR values. [24] To clarify this discrepancy, further investigations including peptide mapping [11] and subunit analysis utilizing enzymatic cleavage to generate Fab segments and Fc segments conjugated with drug-linkers [25] are ongoing. 

Next, SEC analysis of ADCs before and after purification was conducted to measure if the high concentration of NaCl and the use of organic solvent IPA induced aggregation [26]. However, purified and separated ADC DAR species contained less than 3.5% aggregates (Supplementary Materials Figures S13 and S14). Very recently, our research group has completed optimization studies to reduce aggregates by streamlining the conjugation reaction sequence. These advancements will be discussed in future literature.

### 3.2. In Vitro Cell Based Assay of DAR = 1 and DAR = 2 ADCs

**Figure 6:**
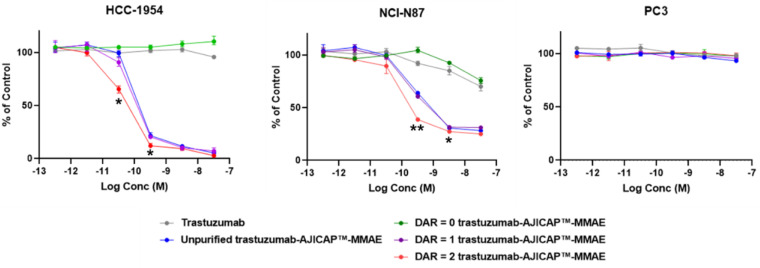
Summary of in vitro cytotoxic activity of purified DAR species: Data shown are mean ± SE from replicate samples (n = 3) for each treatment. Asterisks indicate significant differences compared of DAR = 2 ADC and DAR = 1 ADC at the same conc. point (* *p* < 0.05, ** *p* < 0.01, *t*-test).

Three different cell-based assays were performed to investigate the cytotoxicity of the purified homogeneous DAR compounds. A low HER2 antigen-expressing cell line (PC3: human prostate cancer cell) and HER2 antigen-overexpressing cell lines (NCI-N87: gastric cancer cell and HCC1954: breast cancer cell) were evaluated based on our previous study as shown Figure 6 [10].

Displayed in Table 2, several compounds showed in vitro cytotoxic activity reflective of the DAR against HER2 positive cell lines. In contrast, no cytotoxicity against the HER2 low expressing cell lines was observed. These results indicated that AJICAP-ADCs show both considerable potency and target selectivity toward cancer cells even post preparative HIC purification. The promising cytotoxic assay results described above prompted our research group to plan further biological evaluations and stability assessments of these homogeneous compounds and several studies for homogenous and site-specific ADCs are in progress [27,28,29,30].

## 4. Conclusions

To reduce the heterogeneity of ADCs, a combination strategy of chemical site-specific conjugation and preparative HIC purification was developed. Very recently, Müller and coworkers reported resin screening for the purification of an ADC-mimic [31]. This report indicated that protein recovery in the purification step is dependent on the pH of mobile phase. These reported purification conditions were applied to separate, differentially loaded species of our AJICAP™-ADCs. The mobile phase and resin screening for DAR separation with a site-specific trastuzumab-AJICAP™-MMAE showed that a phenyl-based column and 2 M sodium chloride sufficiently separated the DAR = 1.0 ADC and DAR = 2.0 ADC. The recovery of the ADC after preparative HIC purification was more than 60%, which was relatively higher than the rates in previously reported investigations. Several investigations are underway to reduce the amount of salt necessary in the mobile phase. Further optimizations aimed at enhancement of ADC recovery and amenability to ADC manufacturing are on-going by our research group. Kosmotropic salts such as NaCl and ammonium sulfate may pose a disposal concern in manufacturing facilities and also have potential risk to induce aggregation and precipitation of the ADCs [32].

Chemical conjugation approaches for the site-specific construction of ADCs are of great interest to the oncology therapy community. These methods benefit from straightforward chemistry, manufacturing, and control of the resulting ADCs. Preparative protein chromatography is commonly applied to the purification of biological drugs at a manufacturing-scale. Therefore, the combination of chemical site-specific conjugation and preparative HIC purification can be considered to be a robust strategy to produce next generation ADCs. The feasibility study described herein can serve as a useful guide in the preclinical process development of site-specific and homogenous DAR ADCs. The reported cytotoxic evaluation using three different cell lines in the present manuscript showed that the compounds had considerable potency and target selectivity after purification. The biological and analytical evaluations described herein are the first report regarding the potency of a homogeneous DAR = 1.0 ADC produced by a chemical site-specific approach. We are confident that continued conjugation innovations and strategies in protein purification will aid in the further development of site-specific ADC technologies. Homogeneous DAR = 1.0 ADCs have the potential to be applied to highly potent ADC payloads to overcome the situations of a narrow therapeutic window, and these initial studies provide support for our further biological evaluations to obtain more efficacious ADCs.

## Figures and Tables

**Table 1:** Resin screening for trastuzumab-AJICAP™-MMAE.

Entry	Resin	DAR Separation	% Recovery
1	Phenyl resin (TOYOPEARL Phenyl-650S)	Separated	65%
2	Butyl resin (TOYOPEARL Butyl-650M)	Not eluted	-
3	PPG resin (TOYOPEARL PPG-600M)	Not separated	72%

**Table 2:** IC_50_ Values of trastuzumab-AJICAP™-MMAE species.

Entry	HCC-1954	NCI-N87	PC-3
Trastuzumab	>30 nM	>30 nM	>30 nM
Unpurified trastuzumab-AJICAP™-MMAE	0.21 nM	0.28 nM	>30 nM
Purified site-specifically modified antibody (3, DAR = 0)	>30 nM	>30 nM	>30 nM
Purified DAR = 1 trastuzumab-AJICAP™-MMAE	0.18 nM	0.24 nM	>30 nM
Purified DAR = 2 trastuzumab-AJICAP™-MMAE	0.12 nM	0.12 nM	>30 nM

## Supplementary Materials

The following are available online at https://www.mdpi.com/2073-4468/9/2/16/s1, Figure S1: Full length results of unpurified trastuzumab-AJICAP™-MMAE (4), Figure S2: Full length results of trastuzumab-AJICAP™-MMAE (DAR = 0), Figure S3: Full length results of trastuzumab-AJICAP™-MMAE (DAR = 1), Figure S4: Full length results of trastuzumab-AJICAP™-MMAE (DAR = 2), Figure S5: Deconvoluted spectra of unpurified trastuzumab-AJICAP™-MMAE (4), Figure S6: Deconvoluted spectra of trastuzumab-AJICAP™-MMAE (DAR = 0), Figure S7: Deconvoluted spectra of trastuzumab-AJICAP™-MMAE (DAR = 1), Figure S8: Deconvoluted spectra of trastuzumab-AJICAP™-MMAE (DAR = 2). Figure S9: Full length results of unpurified trastuzumab-AJICAP™-MMAE, Figure S10: Full length results of trastuzumab-AJICAP™-MMAE (DAR = 0), LC 0: light chain, HC 0: heavy chain, Figure S11: Full length results of trastuzumab-AJICAP™-MMAE (DAR = 1) LC 0: light chain, HC 0: heavy chain, HC 1: heavy chain conjugated with one MMAE, Figure S12: Full length results of trastuzumab-AJICAP™-MMAE (DAR = 2); LC 0: light chain, HC 0: heavy chain, HC 1: heavy chain conjugated with one MMAE, Figure S13: Full length results of trastuzumab-AJICAP™-MMAE (DAR = 1), Figure S14: Full length results of trastuzumab-AJICAP™-MMAE (DAR = 2).
