# Supplementary material for: A Purification Strategy Utilizing Hydrophobic Interaction Chromatography to Obtain Homogeneous Species from a Site-Specific Antibody Drug Conjugate Produced by AJICAP™ First Generation
Source: Antibodies (Basel). 2020 May 18;9(2):16. doi: 10.3390/antib9020016 (PMC7344391; doi:10.3390/antib9020016)
Supplement: Supplementary file 1 [file antibodies-09-00016-s001.pdf]

Supporting Information

A Purification Strategy Utilizing Hydrophobic Interaction Chromatography to Obtain Homogeneous Species from a Site-Specific Antibody Drug Conjugate Produced by AJICAP™ First Generation

Yutaka Matsuda,[1] Monica Leung,[1] Tatsuya Okuzumi,[2] Brian A. Mendelsohn*[1]

* Corresponding author.

1: Ajinomoto Bio-Pharma Services, 11040 Roselle Street, San Diego, CA 92121, United States.

2: Ajinomoto Co., Inc., 1-1 Suzuki-cho, Kawasaki, Kanagawa 210-8681, Japan.

Corresponding Author E-mail Address: Brian.Mendelsohn@US.AjiBio-Pharma.com

Full length chromatograms HIC-HPLC S–2
DAR calculator results from Q-TOF MS S–6
Full length chromatograms RP-HPLC S–8
Full length chromatograms SEC-HPLC S–12

Full length chromatograms HIC-HPLC

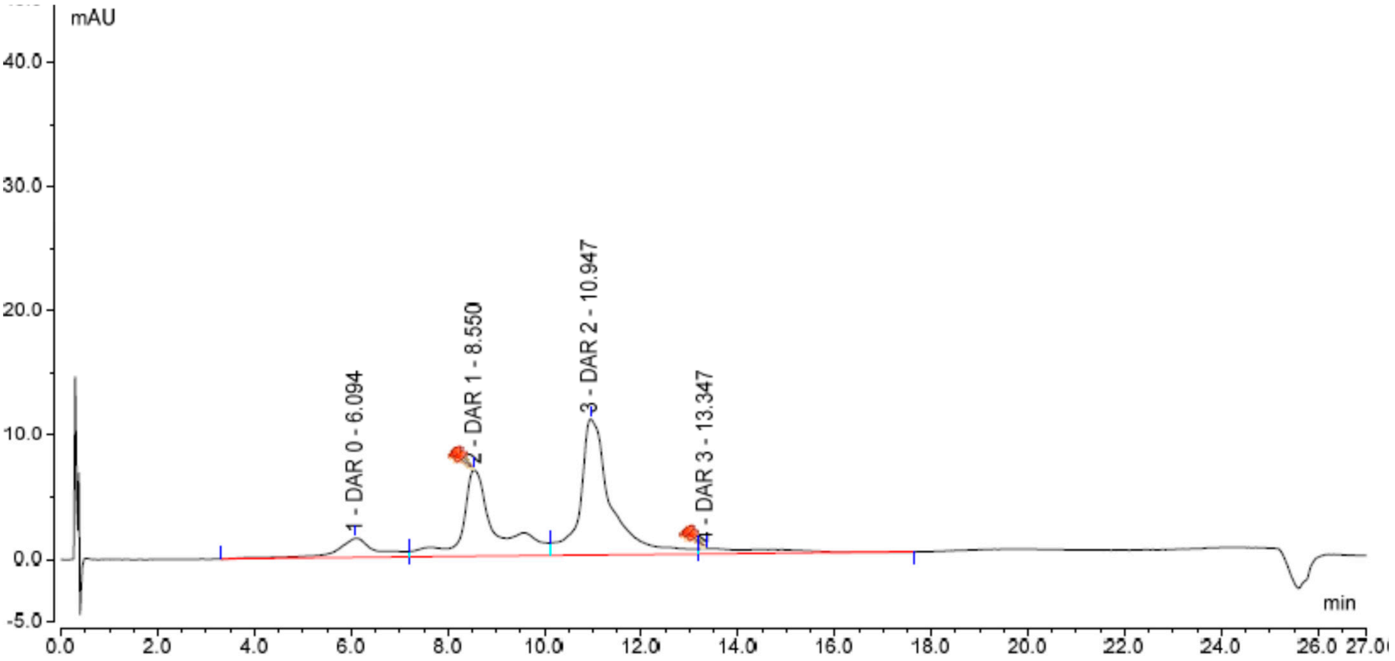

No	Peak time	retention time	Drug load	Relative area %	Weighted peak area
1	DAR = 0	6.09	0	8.28	0
2	DAR = 1	8.55	1	34.01	34.01
3	DAR = 2	10.95	2	51.03	102.06
4	DAR = 3	13.35	3	6.68	20.04
				**Average DAR**	**1.6**

Figure S1. Full length results of unpurified trastuzumab-AJICAP™-MMAE (**4**)

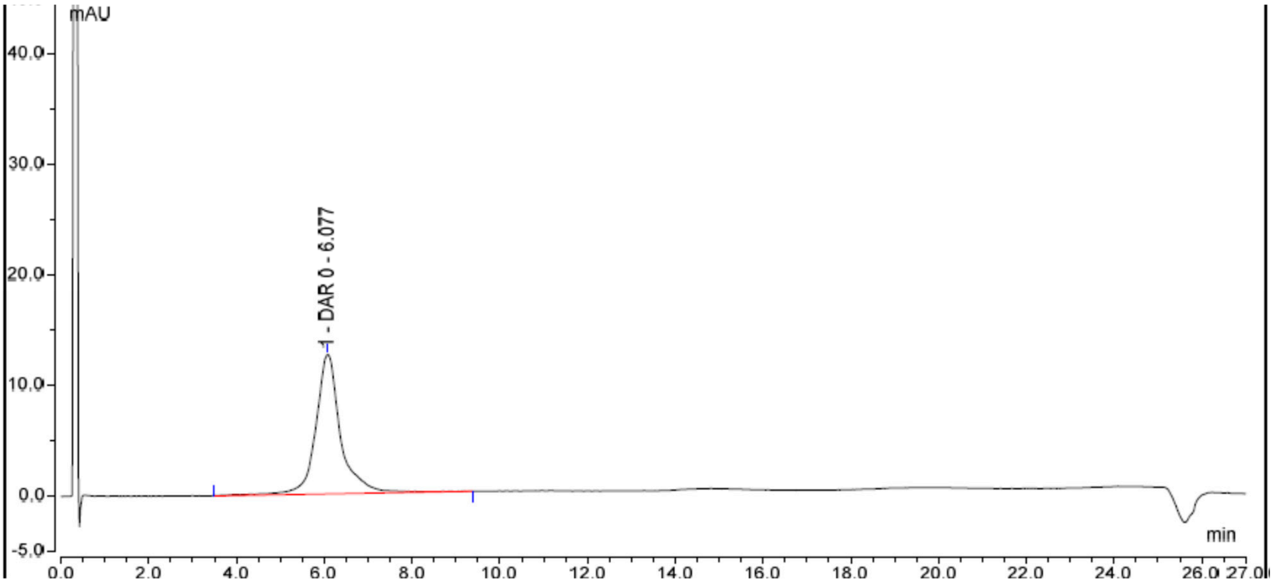

No	Peak time	retention time	Drug load	Relative area %	Weighted peak area
1	DAR = 0	6.08	0	100	0
				**Average DAR**	**0.0**

Figure S2. Full length results of unpurified trastuzumab-AJICAP™-MMAE (**DAR = 0**)

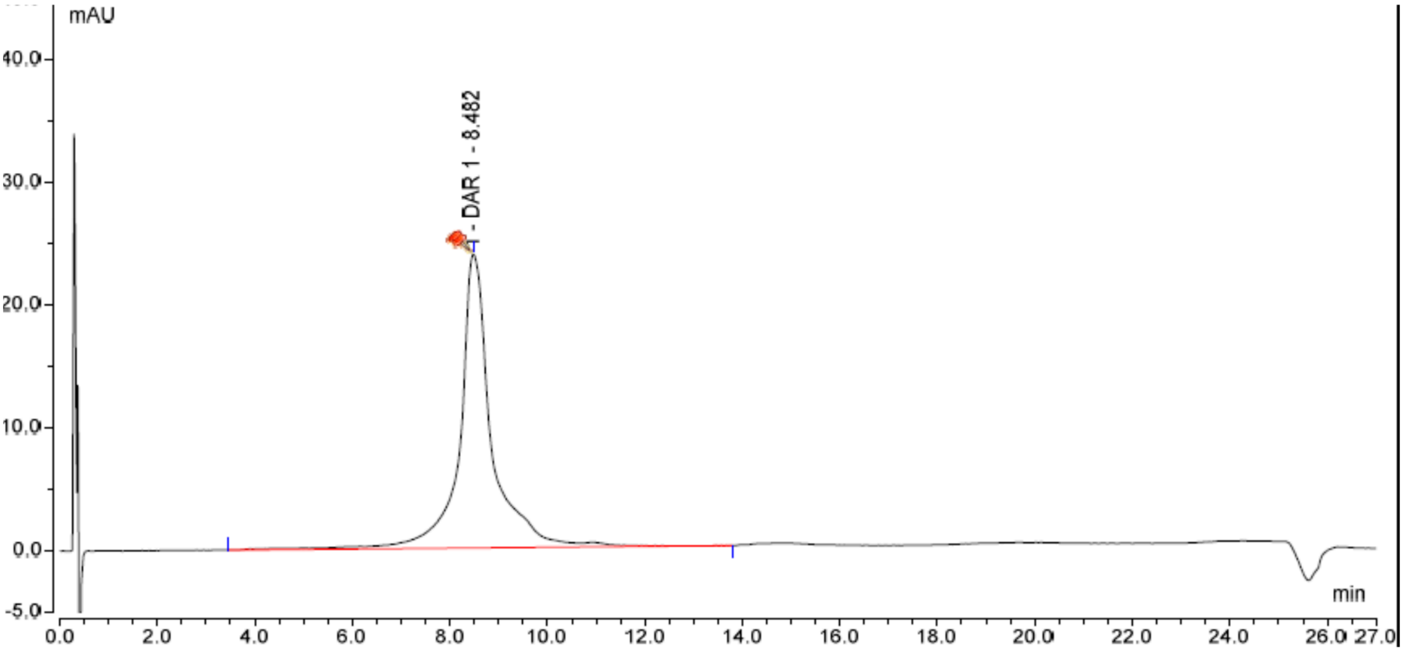

No	Peak time	retention time	Drug load	Relative area %	Weighted peak area
1	DAR = 1	8.48	1	100	1
				**Average DAR**	**1.0**

Figure S3. Full length results of unpurified trastuzumab-AJICAP™-MMAE (**DAR = 1**)

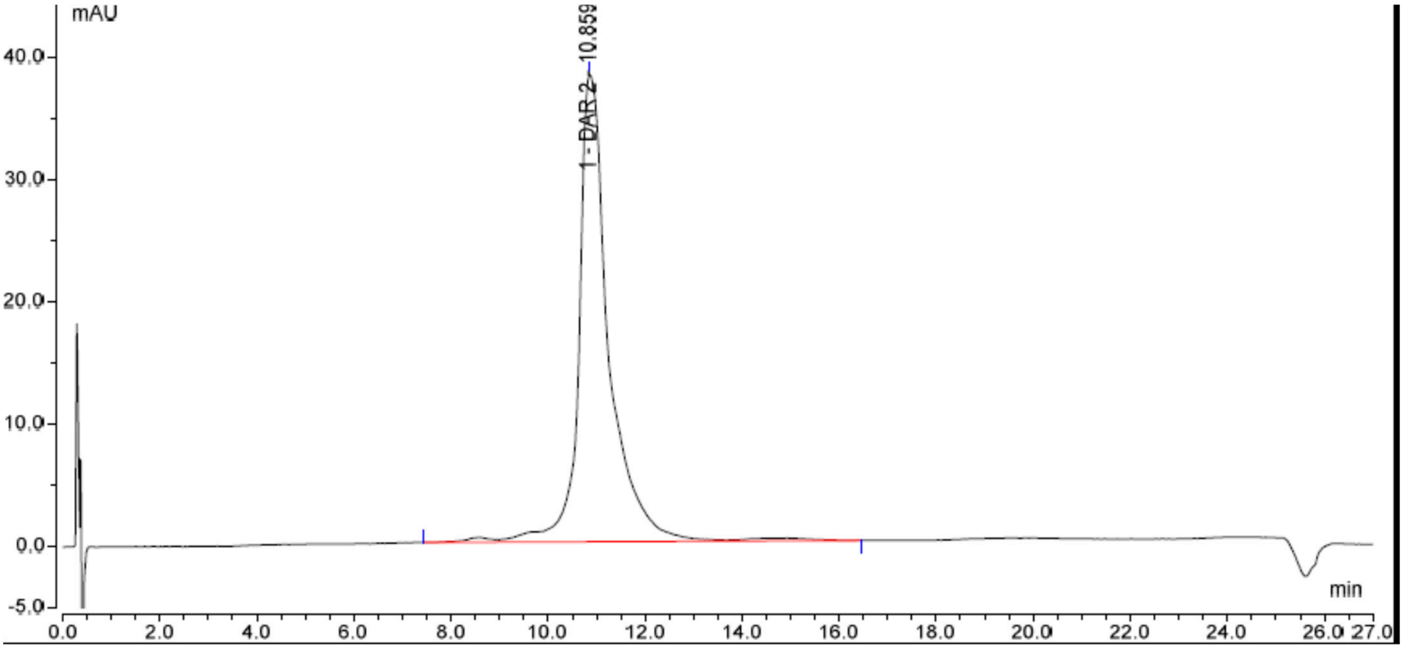

No	Peak time	retention time	Drug load	Relative area %	Weighted peak area
1	DAR = 2	10.86	2	100	2
				**Average DAR**	**2.0**

Figure S4. Full length results of unpurified trastuzumab-AJICAP™-MMAE (**DAR = 2**)

DAR calculation results from Q-TOF MS

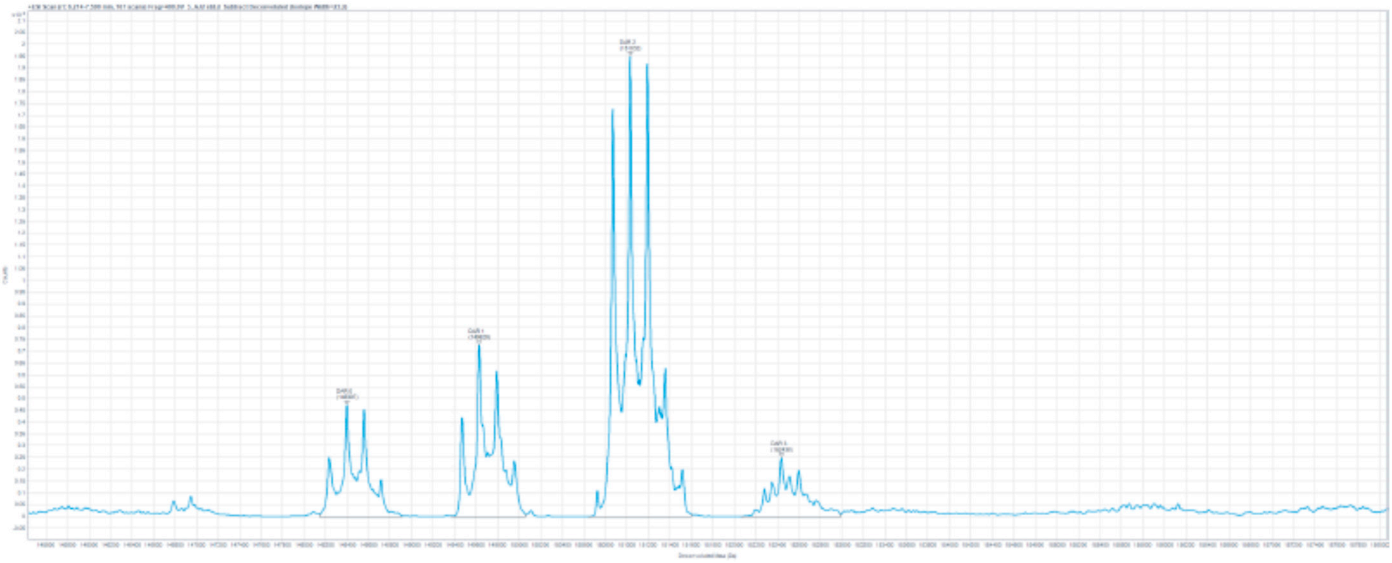

DAR Peak List

DAR Peak	Theoretical Mass (Da)	Observed Mass (Da)	Area	% Area
0	148240	148397	1.01E+006	12.93
1	149644	149629	1.50E+006	19.15
2	151048	151032	4.64E+006	59.40
3	152452	152438	6.65E+005	8.52

Figure S5 Deconvoluted spectra of unpurified trastuzumab-AJICAP™-MMAE (**4**)

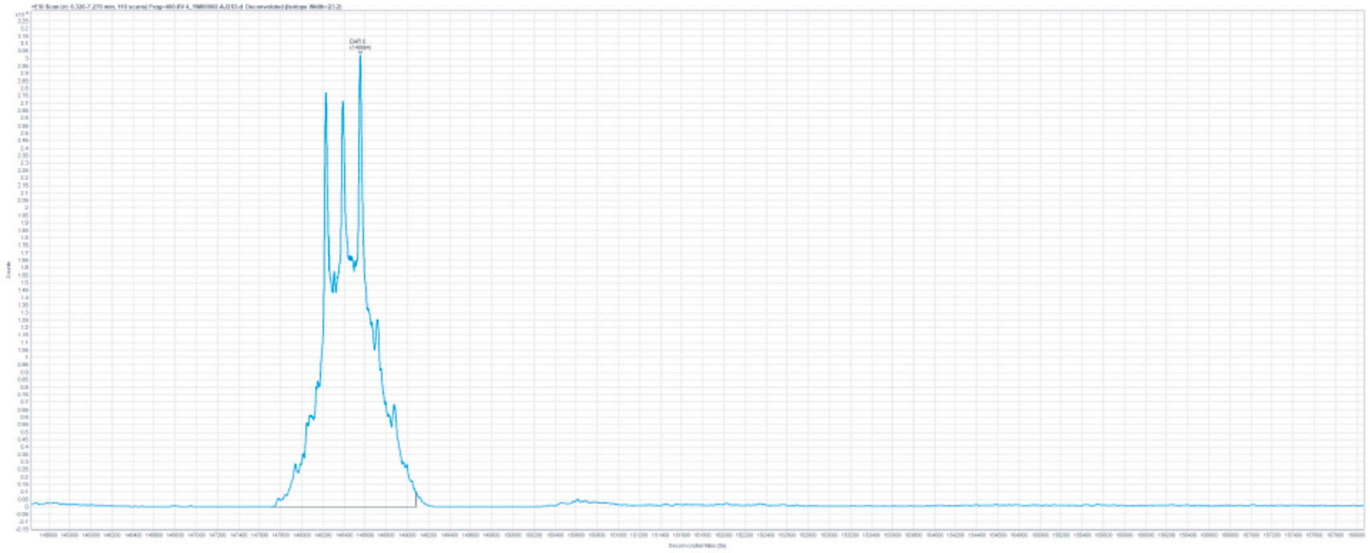

DAR Peak List

DAR Peak	Theoretical Mass (Da)	Observed Mass (Da)	Area	% Area
0	148240	148554	1.23E+007	100.00

Figure S6 Deconvoluted spectra of trastuzumab-AJICAP™-MMAE (**DAR = 0**)

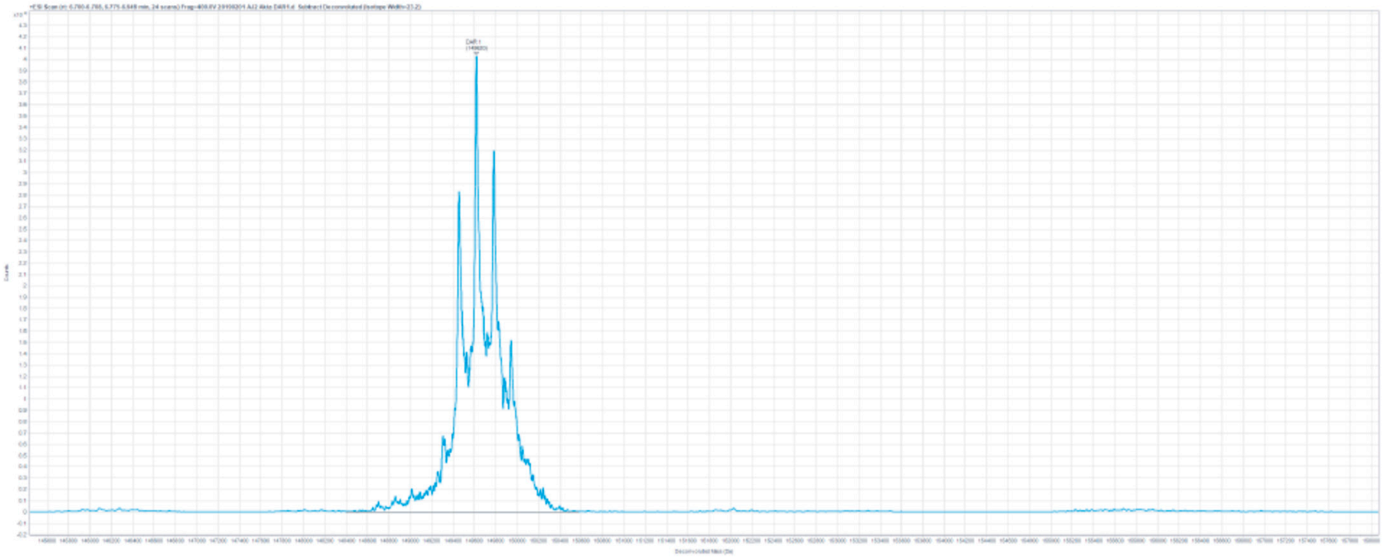

DAR Peak List

DAR Peak	Theoretical Mass (Da)	Observed Mass (Da)	Area	% Area
1	149644	149620	1.22E+007	100.00

Figure S7 Deconvoluted spectra of trastuzumab-AJICAP™-MMAE (**DAR = 1**)

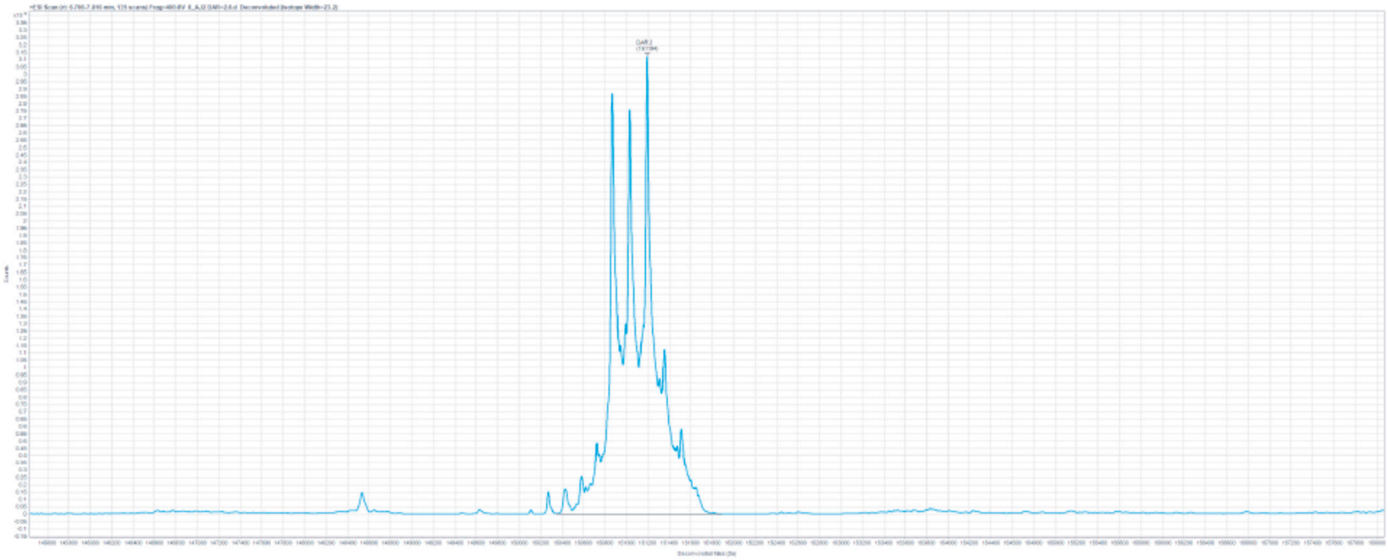

DAR Peak List

DAR Peak	Theoretical Mass (Da)	Observed Mass (Da)	Area	% Area
2	151048	151194	9.99E+006	100.00

Figure S8 Deconvoluted spectra of trastuzumab-AJICAP™-MMAE (**DAR = 2**)

Full length chromatograms RP-HPLC

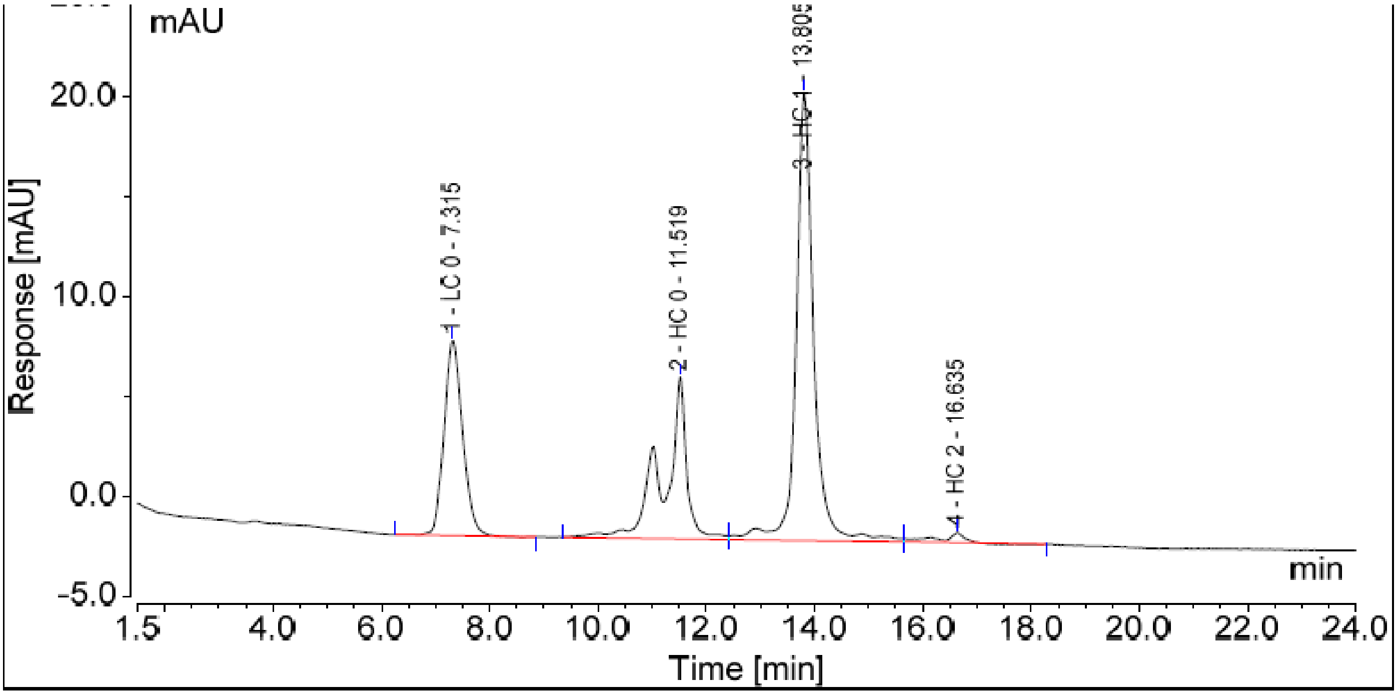

No	Peak time	retention time	Drug load	Relative area %	Weighted peak area
1	LC 0	7.32	0	100	0
2	HC 0	11.52	0	28.24	0
3	HC 1	13.81	1	70.1	70.1
4	HC 2	16.64	2	2.66	5.32
				DAR	**1.5**

Figure S9 Full length results of unpurified trastuzumab-AJICAP™-MMAE (**4**); LC 0: light chain, HC 0: heavy chain, HC 1: heavy chain conjugated with one MMAE, HC 2: heavy chain conjugated with two MMAEs

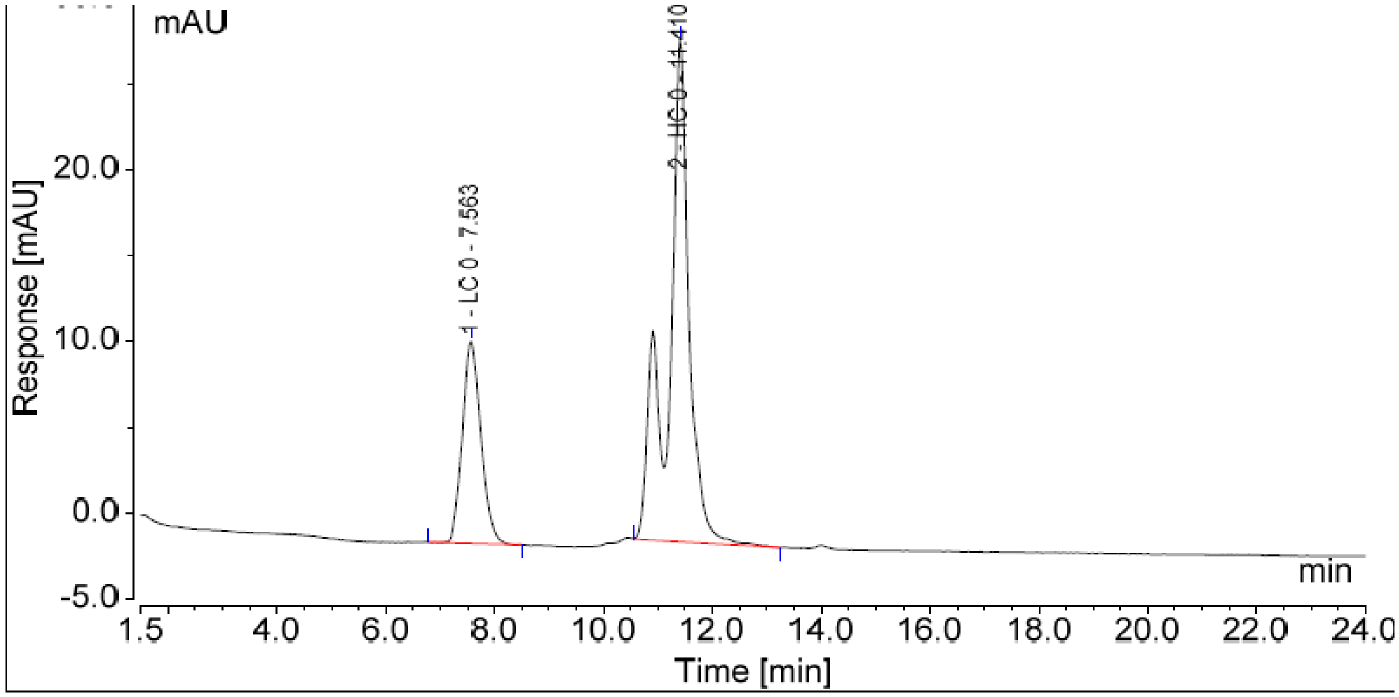

No	Peak time	retention time	Drug load	Relative area %	Weighted peak area
1	LC 0	7.57	0	100	0
2	HC 0	11.41	0	100	0
				DAR	**0.0**

Figure S10 Full length results of trastuzumab-AJICAP™-MMAE (**DAR = 0**), LC 0: light chain, HC 0: heavy chain

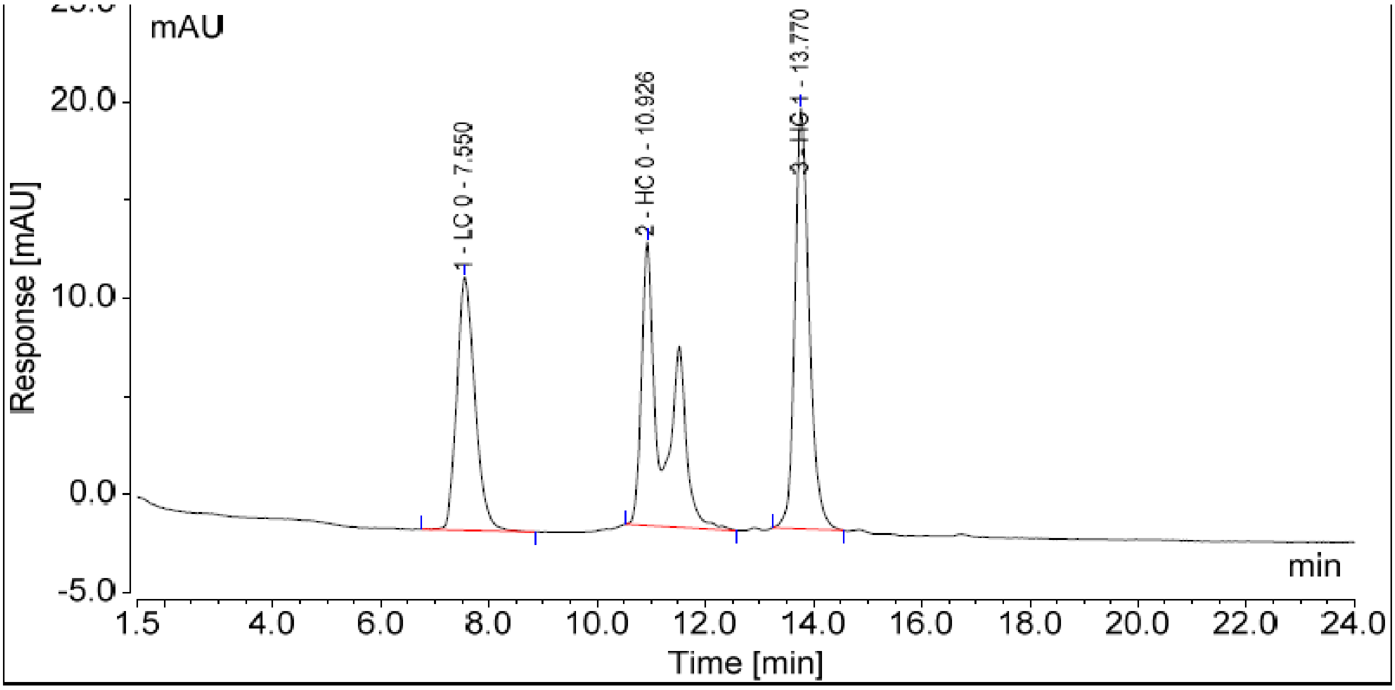

No	Peak time	retention time	Drug load	Relative area %	Weighted peak area
1	LC 0	7.55	0	100	0
2	HC 0	10.93	0	51.14	0
3	HC 1	13.77	1	48.86	48.86
				DAR	**0.98**

Figure S11 Full length results of trastuzumab-AJICAP™-MMAE (**DAR = 1**) LC 0: light chain, HC 0: heavy chain, HC 1: heavy chain conjugated with one MMAE

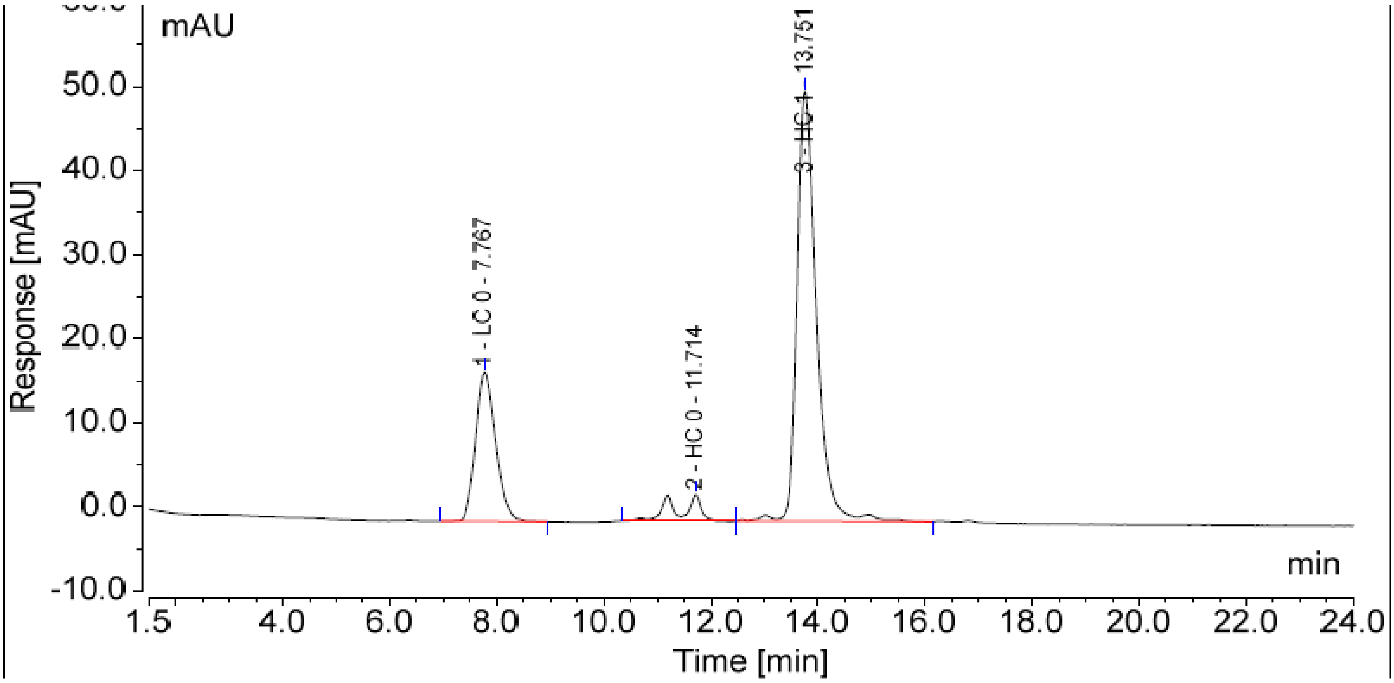

No	Peak time	retention time	Drug load	Relative area %	Weighted peak area
1	LC 0	7.76	0	100	0
2	HC 0	11.71	0	6.79	0
3	HC 1	13.75	1	93.21	93.21
				DAR	**1.9**

Figure S12 Full length results of trastuzumab-AJICAP™-MMAE (**DAR** = **2**); LC 0: light chain, HC 0: heavy chain, HC 1: heavy chain conjugated with one MMAE

Full length chromatograms SEC-HPLC

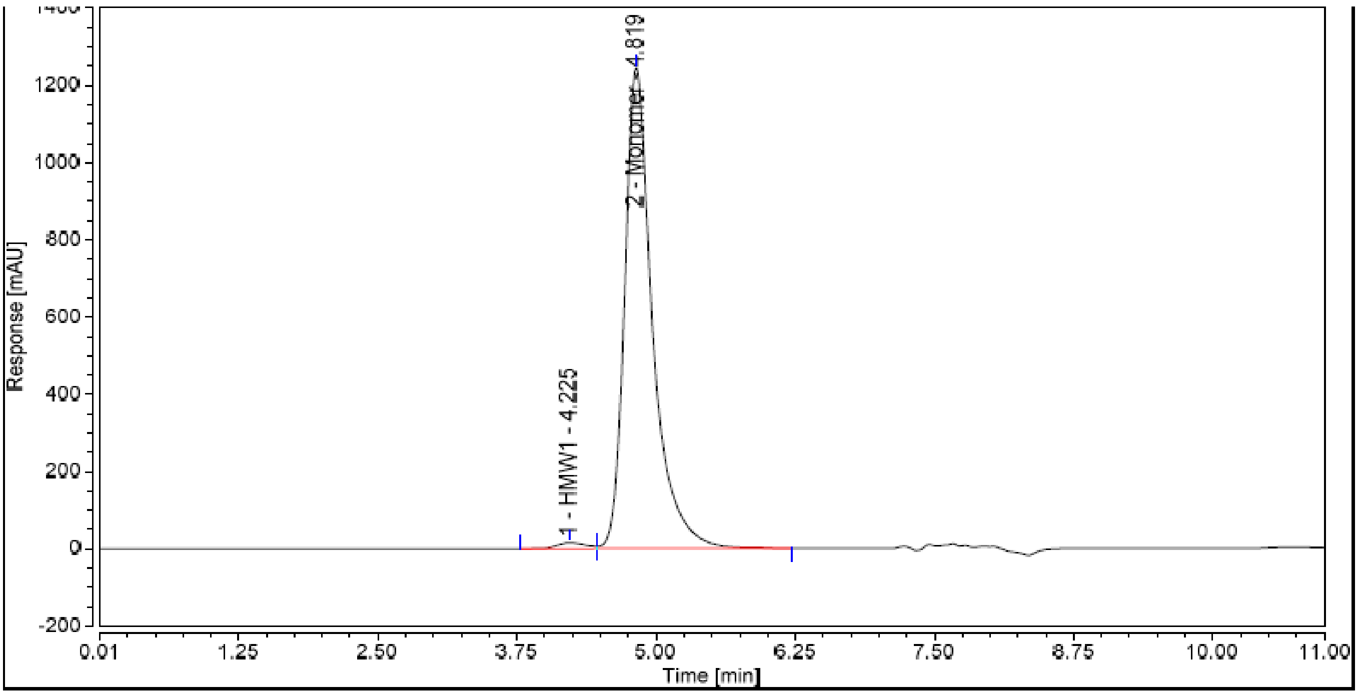

No	Peak time	retention time	Relative area %
1	HWE	4.22	1.79
2	Monomer	4.82	**98.2**

No	Peak time	retention time	Relative area %
1	HWE	4.22	1.79
2	Monomer	4.82	**98.2**

Figure S13. Full length results of trastuzumab-AJICAP™-MMAE (**DAR = 1**)

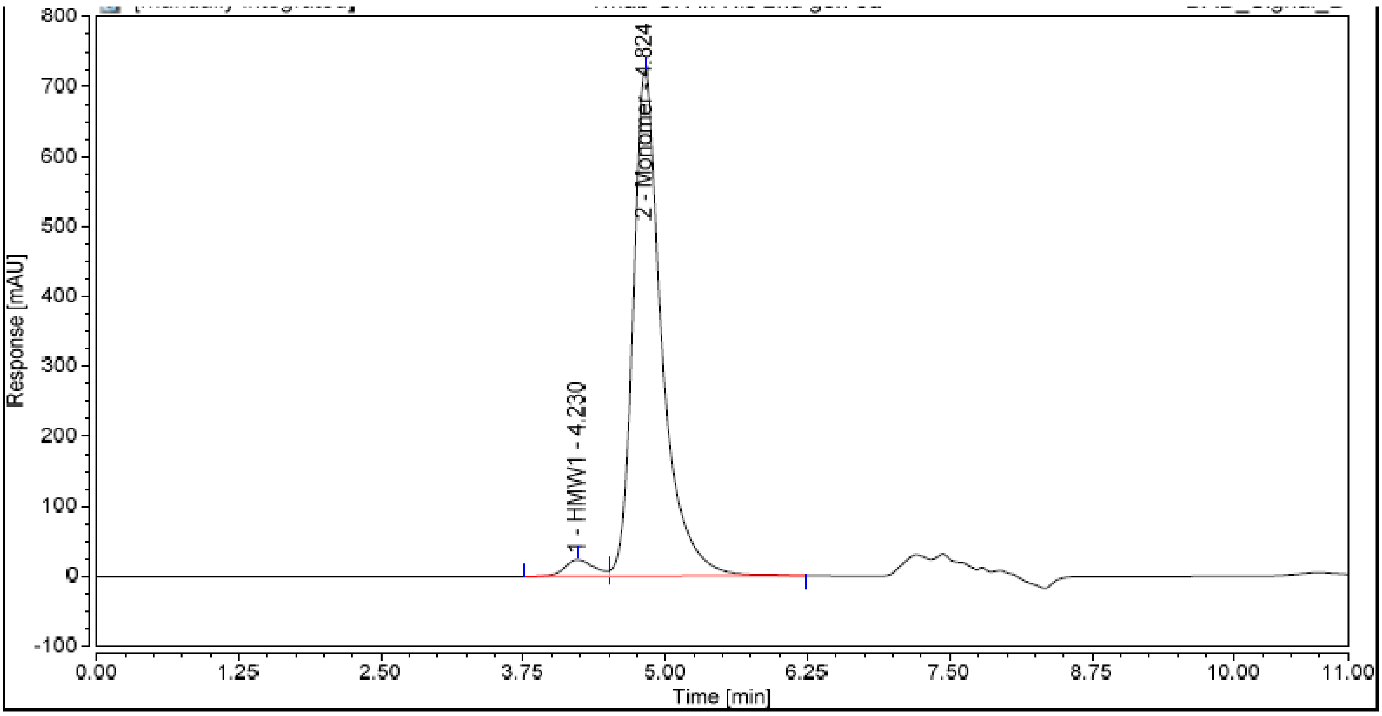

No	Peak time	retention time	Relative area %
1	HWE	4.22	3.40
2	Monomer	4.82	**96.6**

Figure S14. Full length results of trastuzumab-AJICAP™-MMAE (**DAR = 2**)
